# Transplantation of Marginal Organs: Immunological Aspects and Therapeutic Perspectives in Kidney Transplantation

**DOI:** 10.3389/fimmu.2019.03142

**Published:** 2020-01-31

**Authors:** Johan Noble, Thomas Jouve, Paolo Malvezzi, Caner Süsal, Lionel Rostaing

**Affiliations:** ^1^Service de Néphrologie, Hémodialyse, Aphéréses et Transplantation Rénale, Centre Hospitalier Universitaire (CHU) de Grenoble-Alpes, Grenoble, France; ^2^Université Grenoble Alpes, Grenoble, France; ^3^Collaborative Transplant Study, Institute of Immunology, Heidelberg University, Heidelberg, Germany

**Keywords:** kidney transplantation, extended criteria donors, aging, immunosenescence, graft survival

## Abstract

Recent data from the World Population Prospects projects that, by 2050, nearly all regions in the world will have a quarter or more of the population aged 60 and above. Chronic kidney disease (CKD) has a high global prevalence (~13%) worldwide, and the prevalence of chronic kidney disease and end-stage kidney disease increase with age. Kidney transplantation remains the best therapeutic option for end-stage kidney disease, offering a survival benefit in comparison with dialysis maintenance for most patients. This review focuses on immunological aspects of kidney transplantation in older patients and marginal donors, i.e., 60 years or older deceased kidney donors or 50–59 years old deceased kidney donors with comorbidities. Clinical outcomes of kidney recipients in terms of renal and patient survival are more than acceptable even for patients over 70. In this population, the first cause of graft loss is death with a functional graft. However, the inherent issues of these transplantations are the acceptance or refusal of frail kidney from an old donor and the increased immunogenicity of these organs in balance with potential frail and immunosenescent recipients. Finally, the immunosuppressive regimen itself is a challenge for the future of the transplant, to prevent adverse effects such as nephrotoxicity and higher risk of infections or cancer in a population already at risk. Belatacept may have a good place in the immunosuppressive strategy to improve efficacy and the safety posttransplantation.

## Introduction

Chronic kidney disease (CKD) has a high global prevalence worldwide. The prevalence of CKD and end-stage kidney disease (ESKD) increase with age: 27.6% between 60 and 70 years old and 34.3% above 70 years old when taking into account the five stages of CKD (1).

Kidney transplantation is the best therapeutic option for ESKD. Results of kidney transplantation in terms of morbidity and mortality, life quality, and cost effectiveness are better as compared to hemodialysis or peritoneal dialysis (2). However, kidney transplantation, as well as all other solid organ transplantations, is confronted with an organ shortage. To increase the pool of organ donors, the American United Network for Organ Shortage decided to accept organs from Extended Criteria Donors (ECD). The term marginal kidney was replaced by ECD kidney for the first time in 1997 by Kauffman (3). In 2002, a clear definition was given: ECD are defined by deceased donors aged 60 years or older and 50–59 years old deceased donors with at least two of the three following criteria: cerebrovascular cause of death, terminal serum creatinine higher than 1.5 mg/dl (132.6 μmol/L), or history of hypertension (4, 5). Other definitions and aspects of “marginal kidneys” have been studied by different authors such as kidney fibrosis based on histopathology, dual kidney transplantation, donation after cardiac death (DCD), and discarded kidneys (6–9). In 2019, in Europe, ~30% of potential donors are ECD. In North America, ~24% of potential donors are ECD, and nearly 40% of these kidneys are discarded each year (10). ECD kidneys do not follow the classical allocation system of standard kidneys and allow to shorten the time on waiting list at the expense of a better graft (11–13).

## Mechanisms of Organ Aging

Aging has been described as the decline of physiological integrity due to an accumulation of damages, deterioration of proteins, and organelle functions (14). We use the term of senescence to relate biological and functional changes in cells due to aging. Senescence, which is a state of permanent cellular cycle arrest, may occur following a decline over time of cell proliferation capacity as shown by Hayflick (15). Different stimuli may trigger this cellular phenotype such as cells undergoing major DNA damages, telomere dysfunction, and oxidative stress (16). To prevent the risk of malignant transformation, cells may undergo apoptosis, or become senescent. The senescent state is mediated by two cellular pathways: p53/p21 and p16INK4a/pRB pathways (17). This phenotype is also a proinflammatory phenotype, with a high level of inflammatory cytokines and chemokines secretion [e.g., interleukin (IL)-6, IL-8, IL-1]. This induces chronic inflammation in the organs (18).

Senescence in renal cells may be described at different levels using a top–down approach. At a genetic level, Kim et al. described a set of age-related genes (985) in kidneys in 74 healthy patients from 27 to 92 years old (19). Most of these genes showed increased activity and were shared both in the kidney medulla and cortex. Those age-related genes were also shared in other human tissues. These genes involved in kidney aging are for instance the *mortalin-2*, which encodes the heat shock protein 70. Other genes prevent kidney aging, such as the one encoding the *insulin-like growth factor receptor*. However, it is unclear if senescence and age-related genes activations in the organs are genetically or epigenetically inherited. A recent study assessed aging signature in 563 human kidney transcriptomes using next generation RNA sequencing correlated with genomic data and epigenomic data in kidney and non-renal tissues. Finally, the authors identified a total of 19 kidney age-related genes. Five of them were kidney specific (*EDH3, ERP27, MAP4, PPPAR3C*, and *SNX24*). However, these results are preliminary, and to our knowledge, no other team have reproduced this association. Ten of them were associated with biological and clinical signs of aging. Testis-specific Y-like 5 (*TSPYL5*) was the gene with the most significant association with aging (20). *TSPYL5* is one of the nucleosome proteins and plays a role in transcriptional regulation, cell cycle, and probably in cellular senescence (21, 22).

At a molecular level, many mechanisms of kidney aging have been described and well-reported in the review published by López-Otín et al. (14). One of them implies autophagy dysregulation. Autophagy is a physiological process in which cytoplasmic proteins and organelles are non-selectively degraded. Autophagy is critical for terminally differentiated podocytes that are rarely renewed. Autophagy dysregulation results in the accumulation of intracytoplasmic proteins. This eventually results in podocyte degeneration, responsible for age-related glomerulosclerosis and proteinuria (23). Another mechanism of kidney aging is the mitochondrial dysfunction theory causing overproduction of reactive oxygen species, oxidative stress, and age-related damages (24).

At a structural level, aging is related to renal anatomic alterations. Main changes observed in aging kidney are sclerosis (focal and global glomerulosclerosis, tubular atrophy and interstitial fibrosis, arteriosclerosis), nephron hypertrophy, and decline in the number of functional nephrons (25, 26). These modifications lead to renal mass decrease of ~10% per decade and decrease in plasma flow and tubular damages (27). The majority of renal cells are permanently renewed, but podocytes have a limited capacity of regeneration due to their terminally differentiation (28, 29). Podocyte senescence largely contributes to renal aging. The cortex shrinks and the medulla increase in size, with an increased number of renal cysts (30).

At a clinical level, aging leads to glomerular filtration rate (GFR) decline. It has been estimated that, after the fourth decade, a decline of GFR occurs that ranges between 0.63 and 0.75 ml/min/year with kidney aging (26, 31). However, nephrosclerosis and cortical atrophy failed to explain the entirety of the GFR decrease with age (25).

## Immunological Aspect of Aging in Kidney Transplant Recipients

Aging, in the immunological field, is associated with the concept of immunosenescence, which was based on the clinical reports of a higher incidence of infection and cancer and a lower efficacy of vaccination in older people (32). In the field of kidney transplantation, older age of recipients is associated with a lower risk of acute rejection as compared to younger recipients (33). The leading cause of death in old recipient is infection, and death is the leading cause of graft loss (34). Moreover, Mendonça et al. reported a rate of 37.6% of acute rejection in younger recipients (<60 years old) as compared to 22.7% in older (≥60 years old; *p* = 0.01), after a median time of 22 months of follow-up (35). In larger cohorts, it has been shown that the absolute risk of acute rejection decreases for each decade of recipient age (36).

On top of aging, kidney transplant recipients suffer from CKDs and ESKD before transplantation. ESKD itself is associated with a higher risk of infections and virus-related cancers as compared to the general population of the same age. In the general population, the absolute rate of cancer mortality increases with age. However, on the contrary, in kidney transplant patients, the excess risk of cancer-related death decreases with age as compared to the general population. Over 65 years, the absolute risk of cancer-related death is 1.7-fold increased in kidney-transplanted recipients as compared to same age non-transplanted population (37). The mechanism of accelerated immunosenescence in ESKD patients is not clearly understood, but some mechanisms have been assumed: chronic inflammation, oxidative stress, cytomegalovirus (CMV) infection, and epigenetics modifications (38, 39).

The T-cell receptor (TCR) repertoire allows the adaptive immune system to recognize a large number of foreign antigens. The TCR β repertoire is known to decrease almost linearly with age, decreasing from 6.4 × 10^5^ TRBV CDR3 clone types per 10^6^ T cells at the age of 16 years to 3.1 × 10^5^ at the age of 62 years. Although the absolute and relative numbers of total CD3^+^ cells do not differ with age, the percentages of naive CD8^+^ and CD4^+^ cells decrease with age (40). Huang et al. assessed the factors that may accelerate the TCR β repertoire contraction. They showed that age, CMV infection, and ESKD were significantly and independently associated with a shrinking of the TCR β repertoire (41). The impact of age on the TCR β repertoire concerned only the CD8^+^ memory T-cell subset but not the naive T-cell subset.

Other immune cell compartments appear to be affected by aging (42). Impaired B-cells proliferation and antibodies production have been reported. The hypothesis put forward may be IL-2 lower production or T-cell/B-cell interaction dysfunction through CD28 downregulation (43, 44). On contrary, immunosenescence is associated with an increase in cytotoxic natural killer cells capacity with aging. Indeed, some authors reported a decrease in CD56^bright^ subset and an increase in CD56^dim^ subset of natural killer cells, which may play a role in graft antibody-mediated rejection (45).

ESKD also seems to impact the absolute and relative number of different immune cell subsets. Betjes et al. showed that ESKD was associated with a premature immune system aging, i.e., a lower CD31^+^ naive T-cell number as compared to age-matched healthy individuals and a higher percentage of terminally differentiated activated memory CD8^+^ T cells (TEMRA cells) (46). ESKD patients may experience an overinduced apoptosis of naive T cells and an insufficient increase in thymic output and compensating proliferation as compared to same aged healthy individuals (46). Chiu et al. demonstrated how ESKD may accelerate immunosenescence. Indeed, they showed that not only CD8^+^ TEMRA cell frequency was higher in ESKD patients as compared to healthy individuals but also, in multivariate analysis, the level of this senescent phenotype positively correlated with dialysis duration and uremic toxin *p-cresyl* sulfate (47).

Modality of ESKD treatment also impacts immunosenescence as hemodialysis was shown to be associated with a higher level of inflammation as compared to peritoneal dialysis. The chronic inflammation and lymphocyte-sustained activation generated in these patients may accelerate immunosenescence by recruiting new T cells, promote stem cell exhaustion, and explain the lower incidence of observed acute rejection in hemodialysis patients, as compared to peritoneal dialysis patients, before transplantation (39).

The impact of CMV infection on the adaptive immune system homeostasis and immunosenescence is reported in many studies. First, CMV latency is associated with a specific anti-CMV CD8^+^ T-cell repertoire expansion. In healthy donors, using CMV peptides–HLA tetrameric complexes, it has been shown that this subpopulation may reach 10% of CD8^+^ T-cell compartment (48, 49). Posttransplantation, this percentage may reach 18% (50). This unbalanced expansion due to CMV is considered to be detrimental to the immune system of individuals. Similarly, to ESKD, CMV infection, and/or latency are associated with a decrease in naive CD8^+^ T cells and an accumulation of TEMRA cells. Yang et al. showed that a higher anti-CMV IgG level is associated with a lower percentage of total CD4^+^ and CD8^+^ T cells but a higher percentage of CCR7-CD45RA T cells (TEMRA cells) in hemodialysis patients (51). These results were comparable to those found in kidney transplant recipients under immunosuppressive regimen. CMV drives a CD8^+^ T-cell expansion especially CD8^+^CD28 null and TEMRA CD8+ T cells (52).

Finally, in older transplant recipients, Schaenman et al. showed a decreased number of naive CD4^+^ and naive CD8^+^ T cells and an increased number of TEMRA cells and senescent KLRG1^+^ T cells as compared to younger recipients (53).

## Immunological Aspect of Aging in Kidney Transplant Donors

Donor age appears to be an important prognostic factor of long-term outcome after kidney transplantation (54). Nevertheless, the donor age criteria may be misleading when assessed alone (55). In contrast with older recipients, older donors are likely to be more immunogenic. In experimental data, T cells of rats receiving an old graft express a higher level of IFN-γ as compared to those receiving a younger graft. This difference was associated with an accelerated chronic allograft dysfunction (56). de Fijter et al. assessed in a large cohort of kidney transplant recipients the risk factors of acute rejection (57). In a multivariate analysis, donor age ≥50 years old, recipient age <50 years old, and HLA-DR mismatches were significantly associated with a higher risk of acute rejection (risk ratio = 1.53, 1.34, and 2.28 respectively). Interestingly, the risk of acute rejection in older donors was independent of recipient age suggesting other mechanisms than immunosenescence involved.

Aged kidneys have an increased susceptibility to ischemia–reperfusion injury (IRI). The presence of senescent cells in older kidney may result in a reduced tissue regeneration and chronic low level of inflammation. Different mechanisms may explain the reduced tolerance to IRI: impairment of mitochondrial functions which results in a decrease in antioxidant defenses, reduced expression of heat shock protein-70 involved in transmitochondrial transport, and telomere shortening contributing to the increase in the process of senescence (58). Conversely, IRI like hypertension was shown to increase the level of senescence in donor kidney (59).

In the end, the increased level of inflammation and edema induced by IRI in aged kidneys is the root of a stronger immune response. Indeed, antigen-presenting capacities of dendritic cells seem to increase with age (60). Nevertheless, regarding dendritic cell functions in aging, little is known currently and data in the literature are controversial (61, 62). Moreover, it was shown that, after acute tubular necrosis, there is an increased expression of HLA molecules in tubular cells and accumulation of inflammatory cells (63). Clinically, delayed graft function induced by IRI is associated with a 38% increased risk of acute rejection (64). The impact of IRI on ECD kidneys is significant, and those kidney benefit from machine of perfusion with a lower rate of delayed graft function and higher kidney survival rate as compared to cold storage (65, 66).

## Clinical Results in Recipients of Marginal Kidneys

Since the proportions of older patients on the waiting list and ECD have largely increased, many studies assessed the benefit of kidney transplantation in these populations. First, transplantation with kidney from ECD has been associated with a higher survival rate as compared to maintenance of the waiting list in >60 years old recipients (67). In this European study, the 5-year survival rate was 83.6% for recipients of ECD kidney as compared to 67.4% for patients who remained on the waiting list. Recipient's age was the major predictive risk factor of mortality in the early- and late-period posttransplantation with time on dialysis before transplantation and diabetes mellitus (68, 69).

Only few studies assessed the long-term results of recipients receiving a kidney graft from ECD as compared to standard criteria donors (SCDs) (70). In 2015, Aubert et al. assessed the long-term results of graft survival between ECD and SCD in 2,763 recipients in a French cohort and in a validation cohort. ECD was associated with a lower graft survival [hazard ratio (HR) = 1.87 (1.50–2.32), *p* < 0.001] as compared to SCD at 7 years posttransplantation. In the multivariate Cox analysis, ECD, cold ischemia, and presence of donor-specific alloantibodies (DSA) at transplantation were significantly associated with kidney allograft loss. The model was adjusted on donor type (deceased vs. living), presence of diabetes in donor, graft rank, and number of HLA-A/B/DR mismatches (71). Recipients of ECD with circulating DSAs at the time of transplantation had the worse kidney graft outcome with a 4.4-fold increased risk of graft loss as compared to those without DSA.

In 2016, Querard et al. conducted a meta-analysis to assess the results of ECD transplantation. From 29 studies, they estimated the non-adjusted pooled risk ratio of patient survival at 5 years at 1.62 (1.18–2.22) and of death-censored graft loss at 1.69 (1.18–2.34) in favor of SCD as compared to ECD (72). The results largely came from North America studies. Moreover, only a very small number of studies were adjusted with usual confounders. In Europe, the non-adjusted pooled risk ratios were lower than in North America.

Van Ittersum et al. published the results of 3,062 kidney recipients after 7.8 years of follow-up in a European population (73). Six hundred nineteen recipients received an ECD kidney, and 2,443 received a SCD kidney. Recipients from deceased ECD donors had a higher risk of death-censored graft failure [HR = 1.92 (1.63–2.26)] and death [HR = 1.45 (1.26–1.67)] as compared to other recipients (deceased donors with SCD criteria and living donors). At 10 years, ECD criteria was associated with an absolute risk of 16.9% for graft lost and 10.1% for death, as compared to SCD. In a subgroup analysis of recipients of the same study, DCD with ECD criteria had the lower graft and patient survival prognosis. Tomita et al. specifically studied ECD after DCD and did not find an increased overall risk of graft loss as compared to SCD. However, the risk of death-censored graft loss was higher in older ECD and donors with an history of hypertension or cerebrovascular events (74).

However, some published data report excellent results with ECD transplantation as compared to SCD. In the study of Palkoci et al. 50 ECD were compared to 107 ECD kidney recipients. At 1 year, the rate of acute rejection was not statistically different, and at 5 years, the death-censored survival rate was not different (92%, *P* = 0.884) in both groups (75). Another study conducted by Kim et al., which included 42 ECD and 364 SCD, showed higher serum creatinine level at 12 months in ECD, but the survival rate was similar as compared to SCD (76).

## Therapeutic Strategies in ECD Kidney Transplantation

Different immunosuppressive strategies in ECD recipients may be discussed (Table 1). The goal in ECD is to reduce not only the incidence of infections and cancers but also acute rejection in this at-risk population. In induction therapy, rabbit antithymocyte globulin (rATG) has shown lower risk of acute rejection as compared to IL-2 receptor antagonists without an increased risk of death in older recipients and high-risk kidney such as ECD (86). Steroids maintenance or withdrawal has to be weighed between the higher risk of acute rejection and the risk of side effects in older patients. It was shown that an early steroid withdrawal at the time of first discharge posttransplantation was associated with a better adjusted overall graft survival [HR = 1.32 (1.1–1.56), *P* = 0.002) and patient survival [HR = 1.46 (1.16–1.83), *P* = 0.001] but not death-censored graft survival. In a subgroup analysis, these results were confirmed only in the T-cell-depleting induction treatment (thymoglobulin) group but not in the IL-2 receptor blocker (Basiliximab) group (87).

**Table 1:** Study characteristics of trials evaluating immunosuppressive regimen in expanded criteria donors.

	**Reference**	**Study design**	**Results**
Induction	Gill et al. (77)	Retrospective. rATG or IL2RA or alemtuzumab 14,820 patients	rATG > IL2RA/alemtuzumab in terms of rejection rate and graft survival in high-risk patients
Steroid withdrawal	Aull et al. (78)	Retrospective. 634 patients. 46% ECD	At 5 years: 90.2% patient survival 87.6% DCGS 12.8% acute rejection
	Segolini et al. (79)	88 ECD IL2R + MMF + tacrolimus and steroid reducing or withdrawal	At 3 years: 13.6% rejection rate At 4 years: 96% patient survival 79% graft survival
Belatacept	Durrbach et al. (80) (BENEFIT-EXT)	Prospective. 543 patients. Belatacept vs. CsA IL2RA + MMF + steroids	At 7 years: 73 vs. 78% patient survival 88 vs. 81% DCGS 21 vs. 17% acute rejection
Delayed CNI	Stratta et al. (81)	Prospective. 101 ECD. ATG or Alemtuzumab + MMF + steroids	At 4 years: 12% acute rejection 93% patient survival 83% graft survival
	Arbogast et al. (82)	Prospective. 89 ECD. rATG + MMF + steroids.	At 5 years: 24% acute rejection 88% patient survival 70% graft survival
mTOR inhibitors	Furian et al. (83)	Comparative non-randomized. 31 ECD. rATG + Sirolimus + MMF + steroids	At 1 year: 19% acute rejection 100% patient survival 97% graft survival
	Cruzado et al. (84)	Comparative non-randomized. 42 ECD. rATG + Sirolimus + MMF + Steroids	At 3 years: 8% acute rejection 76% patient survival 90% DCGS
	Ferreira et al. (85)	Prospective randomized. 171 ECD. rATG + tacrolimus + everolimus + steroids vs. MMF	At 1 year: 95 vs. 84% avute rejection 89 vs. 99% DCGS 90 vs. 99% patient survival

*rATG, rabbit antithymoglobulin; IL2RA, IL2 receptor antagonist; ECD, expanded criteria donors; MMF, mycophenolate mofetil; CsA, cyclosporin A; DCGS, death-censored graft survival*.

In the field of kidney transplantation, clinicians seek intensively for new immunosuppressive regimens to avoid calcineurin inhibitors (CNIs) nephrotoxicity. In 2011, the US Food and Drug Administration approved the use of belatacept. This drug is a fusion protein that bind CD80/86 onto antigen-presenting cells and thereby blocks effector T cells by preventing interactions with CD28 (88). In the BENEFIT-EXT trial, 543 ECD recipients received either cyclosporine- or belatacept-based regimen (80). At 7 years posttransplantation, mean estimated GFR was 53.9 ± 1.9, 54.2 ± 1.9, and 35.3 ± 2.0 ml/min per 1.73 m^2^ for belatacept more intensive, belatacept less intensive, and cyclosporine groups, respectively (*P* < 0.001). This showed the benefit of avoiding CNI nephrotoxicity in those kidneys. Death-censored graft loss and patient survival was similar in all groups except for a higher incidence of posttransplant lymphoproliferative disorders in EBV-negative recipients treated by belatacept. Posttransplantation switch from CNI to belatacept within the first 6 months also seems efficient to improve renal graft function from ECD (89). Mammalian target of rapamycin (mTOR) inhibitors may also be a valuable option to avoid CNI nephrotoxicity, but large randomized and controlled studies are missing (90). Most of non-randomized studies showed acceptable results of graft survival and rejection rate with sirolimus or everolimus in CNI minimization strategies (86). Yet, the benefit of mTOR inhibitors as compared to mycophenolate in ECD patients is controversial (91). Indeed, despite a lower incidence of CMV infection/disease, Ferreira et al. study was prematurely terminated due to a higher incidence of acute rejection, graft loss, and death in the mTOR inhibitor group, i.e., tacrolimus + everolimus as compared to tacrolimus + MPA (85).

Despite all these clinical results of ECD vs. SCD, kidney transplantation with ECD remains a valuable option. Indeed, in North America, Ojo et al. showed that ECD transplantation improve patient survival over maintenance dialysis treatment with an increase of 5 years in life expectancy (92). These results were consistent in the European population (67).

## Risk Stratification

In 2009, Rao et al. published the kidney donor risk index based on the Scientific Registry of Transplant recipients in the North American population (93). The kidney donor risk index appears to be an interesting tool to stratify the risk and estimate outcomes posttransplantation based on 14 donor and transplant factors associated with death and graft failure. This score is currently used in the United States to allocate kidney graft for single kidney transplantation or dual kidney transplantation (94). KDPI score was assessed also in European cohorts of high-risk donor–recipient pairs and was efficient to improve the graft outcome prediction (95).

In Europe, the Eurotransplant senior program (ESP) was created to improve transplant allocation and shorten the time on waiting list. It was designed to allocate kidney from ≥65 years old donors to ≥65 years old recipients regardless of HLA matching but with a focus on reducing the cold ischemia time (96). Frei et al. published the 5-year results of the ESP and showed that death-censored graft survival of ESP patients was similar when compared to old donor giving to other any recipients (67% survival) but was lower as compared to any aged donor giving to old recipients (81%). These results were obtained at the price of higher incidence of acute rejection (97). Results from the Dutch Organ Transplant Registry, which is part of Eurotransplant and ESP, showed a 5-year death censored graft survival of 83.8% in DBD and 75.3% in DCD (98). In this old recipient population, delayed graft function was a strong risk factor of death (+40% risk) and of rejection (+57%) and DSA development (99).

## Conclusions and Perspectives

Kidney transplantation of “marginal donors” to old recipients implies different specificities: immunosenescence of recipients and higher risk of complications (i.e., infections and cancers), higher immunogenic response of older kidneys and increased susceptibility to IRI, and worse outcome than SCD kidneys. Nevertheless, older patients still benefit from transplantation rather than remaining in the waiting list. New immunosuppressive regimens and strategies such as costimulation blockade, early steroids withdrawal, and CNI minimization strategies may be useful to improve patient and renal outcomes in ECD recipients. The goal in the future will be to minimize CNI-associated toxicity such as nephrotoxicity, cardiovascular morbidity and mortality, and malignancy in the particular population of ECD recipients. To achieve this goal, we need to improve the risk stratification before clinicians allocate a kidney from ECD to an old recipient. New randomized studies need to be done in ECD transplantation.

## Author Contributions

JN performed most of the literature search and wrote the manuscript. TJ, PM, and CS contributed to the literature search and carefully read the manuscript. LR finalized the manuscript.

### Conflict of Interest

The authors declare that the review was conducted in the absence of any commercial or financial relationships that could be construed as a potential conflict of interest.
